# (4*S*,5*S*)-2-(3-Methoxy­phen­yl)-1,3-dioxolane-4,5-dicarboxamide

**DOI:** 10.1107/S1600536809032991

**Published:** 2009-08-26

**Authors:** De-Cai Wang, Tao Ge, Wen-Yuan Wu, Wei Xu, Zheng Yang

**Affiliations:** a State Key Laboratory of Materials-Oriented Chemical Engineering, College of Life Science and Pharmaceutical Engineering, Nanjing University of Technology, Xinmofan Road No. 5 Nanjing, Nanjing 210009, People’s Republic of China; b Department of Applied Chemistry, College of Science, Nanjing University of Technology, Xinmofan Road No. 5 Nanjing, Nanjing 210009, People’s Republic of China

## Abstract

In the title compound, C_12_H_14_N_2_O_5_, the five-membered ring adopts an envelope conformation. In the crystal structure, inter­molecular N—H⋯O inter­actions link the mol­ecules into a three-dimensional network. A weak C—H⋯π inter­action is also found.

## Related literature

For general background, see: Kim *et al.* (1994 ▶); Pandey *et al.* (1997 ▶). For bond-length data, see: Allen *et al.* (1987 ▶).
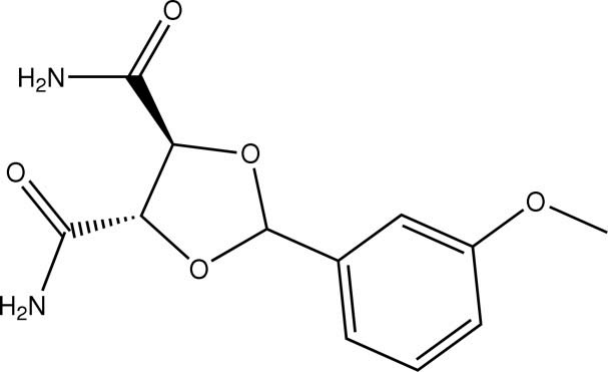


## Experimental

### 

#### Crystal data

C_12_H_14_N_2_O_5_
*M* *_r_* = 266.25
Orthorhombic, 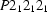

*a* = 9.2340 (18) Å
*b* = 9.852 (2) Å
*c* = 14.266 (3) Å
*V* = 1297.8 (5) Å^3^
*Z* = 4
Mo *K*α radiation
μ = 0.11 mm^−1^
*T* = 294 K
0.30 × 0.20 × 0.20 mm

#### Data collection

Enraf–Nonius CAD-4 diffractometer
Absorption correction: ψ scan (North *et al.*, 1968 ▶) *T*_min_ = 0.971, *T*_max_ = 0.979
2599 measured reflections
1378 independent reflections
1157 reflections with *I* > 2σ(*I*)
*R*_int_ = 0.027
3 standard reflections frequency: 120 min intensity decay: 1%

#### Refinement

*R*[*F* ^2^ > 2σ(*F* ^2^)] = 0.050
*wR*(*F* ^2^) = 0.132
*S* = 1.33
1378 reflections
170 parameters
H-atom parameters constrained
Δρ_max_ = 0.31 e Å^−3^
Δρ_min_ = −0.40 e Å^−3^

### 

Data collection: *CAD-4 Software* (Enraf–Nonius, 1989 ▶); cell refinement: *CAD-4 Software*; data reduction: *XCAD4* (Harms & Wocadlo, 1995 ▶); program(s) used to solve structure: *SHELXTL* (Sheldrick, 2008 ▶); program(s) used to refine structure: *SHELXTL*; molecular graphics: *SHELXTL*; software used to prepare material for publication: *SHELXTL* and *PLATON* (Spek, 2009 ▶).

## Supplementary Material

Crystal structure: contains datablocks global, I. DOI: 10.1107/S1600536809032991/hk2731sup1.cif

Structure factors: contains datablocks I. DOI: 10.1107/S1600536809032991/hk2731Isup2.hkl

Additional supplementary materials:  crystallographic information; 3D view; checkCIF report

## Figures and Tables

**Table 1:** Hydrogen-bond geometry (Å, °)

*D*—H⋯*A*	*D*—H	H⋯*A*	*D*⋯*A*	*D*—H⋯*A*
N2—H2*B*⋯O5^i^	0.86	2.33	3.076 (4)	145
N2—H2*A*⋯O4^i^	0.86	2.13	2.926 (4)	153
N1—H1*B*⋯O2^ii^	0.86	2.31	3.045 (4)	143
N1—H1*A*⋯O5^iii^	0.86	2.20	2.952 (4)	146
C9—H9*A*⋯*Cg*1^iv^	0.98	2.82	3.640 (4)	141

Symmetry codes: (i) 
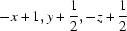
; (ii) 
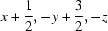
; (iii) 
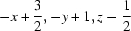
; (iv) 
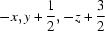
. *Cg*1 is the centroid of the C2–C7 ring.

**Fig. 1.**
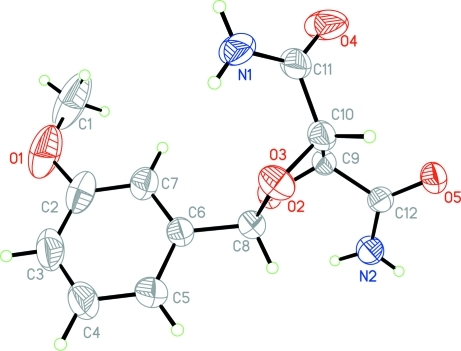
The molecular structure of the title molecule with the atom-numbering scheme. Displacement ellipsoids are drawn at the 30% probability level

**Fig. 2.**
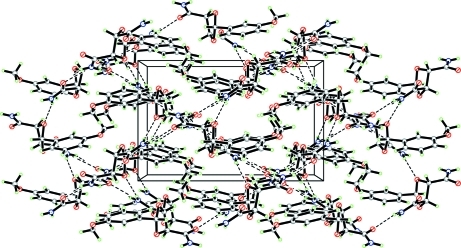
A partial packing diagram. Hydrogen bonds are shown as dashed lines.

## supplementary crystallographic information

### Comment

Antitumor platinum drug is one kind of the most effective anticancer agents currently available. (2*S*,3S)-Diethyl 2,3-*O*-alkyltartrate analogues are the starting materials for the syntheses of platinum complexes with antitumor activity (Kim *et al.*, 1994) and are also important intermediates in organic syntheses (Pandey *et al.*, 1997). As part of our studies on the syntheses and characterizations of these compounds, we report herein the crystal structure of the title compound.

In the molecule of the title compound, (Fig. 1), the bond lengths (Allen *et al.*,  1987) and angles are within normal ranges. Ring A (C2-C7) is, of course, planar, while ring B (O2/O3/C8-C10) adopts envelope conformation with atom O2 displaced by 0.456 (3) Å from the plane of the other ring atoms.

In the crystal structure, intermolecular N-H···O interactions (Table 1) link the molecules into a three-dimensional network (Fig. 2), in which they may be effective in the stabilization of the structure. A weak C—H···π interaction (Table 1) is also found.

### Experimental

For the preparation of the title compound, a mixture of (2S,3S)-diethyl- tartrate (500 mg, 2.43 mmol), 3-methoxybenzaldehyde (331 mg, 2.43 mmol), anhydrous copper(II) sulfate (776 mg, 2.86 mmol) and one drop of methanesulfonic acid in anhydrous toluene (8 ml) was stirred at room temperature for 8 h. Anhydrous magnesium sulfate (30 mg) was added to the reaction mixture, which was then stirred for 20 min. Then, the resulting colorless precipitate was obtained by evaporation and dried in the vacuo (yield; 83%). The obtained colorless product (654 mg, 2 mmol) was dissolved in anhydrous ethanol (40 ml), and a current of dry ammonia, dried by calcium cholride was passed into the reaction mixture at room temperature for 4 h. Then, the reaction mixture was filtered and the resulting product was evaporated to dryness. Crystals suitable for X-ray analysis were obtained by slow evaporation of a methanol solution after four weeks.

### Refinement

H atoms were positioned geometrically with N-H = 0.86 Å (for NH_2_) and C-H = 0.93, 0.98 and 0.96 Å for aromatic, methine and methyl H atoms, respectively, and constrained to ride on their parent atoms, with U_iso_(H) = xU_eq_(C,N), where x = 1.5 for methyl H, and x = 1.2 for all other H atoms. The absolute structure could not be determined reliably, and 986 Friedel pairs were averaged before the last cycle of refinement.

### Crystal data

**Table:**

C_12_H_14_N_2_O_5_	*F*(000) = 560
*M**_r_* = 266.25	*D*_x_ = 1.363 Mg m^−^^3^
Orthorhombic, *P*2_1_2_1_2_1_	Mo *K*α radiation, λ = 0.71073 Å
Hall symbol: P 2ac 2ab	Cell parameters from 25 reflections
*a* = 9.2340 (18) Å	θ = 9–13°
*b* = 9.852 (2) Å	µ = 0.11 mm^−^^1^
*c* = 14.266 (3) Å	*T* = 294 K
*V* = 1297.8 (5) Å^3^	Block, colorless
*Z* = 4	0.30 × 0.20 × 0.20 mm

### Data collection

**Table:**

Enraf–Nonius CAD-4 diffractometer	1157 reflections with *I* > 2σ(*I*)
Radiation source: fine-focus sealed tube	*R*_int_ = 0.027
graphite	θ_max_ = 25.3°, θ_min_ = 2.5°
ω/2θ scans	*h* = −11→0
Absorption correction: ψ scan (North *et al.*, 1968)	*k* = −11→11
*T*_min_ = 0.971, *T*_max_ = 0.979	*l* = −17→0
2599 measured reflections	3 standard reflections every 120 min
1378 independent reflections	intensity decay: 1%

### Refinement

**Table:**

Refinement on *F*^2^	Primary atom site location: structure-invariant direct methods
Least-squares matrix: full	Secondary atom site location: difference Fourier map
*R*[*F*^2^ > 2σ(*F*^2^)] = 0.050	Hydrogen site location: inferred from neighbouring sites
*wR*(*F*^2^) = 0.132	H-atom parameters constrained
*S* = 1.33	*w* = 1/[σ^2^(*F*_o_^2^) + (0.0632*P*)^2^ + 0.0799*P*] where *P* = (*F*_o_^2^ + 2*F*_c_^2^)/3
1378 reflections	(Δ/σ)_max_ < 0.001
170 parameters	Δρ_max_ = 0.31 e Å^−^^3^
0 restraints	Δρ_min_ = −0.40 e Å^−^^3^

### Special details

**Table:**

Geometry. All e.s.d.'s (except the e.s.d. in the dihedral angle between two l.s. planes) are estimated using the full covariance matrix. The cell e.s.d.'s are taken into account individually in the estimation of e.s.d.'s in distances, angles and torsion angles; correlations between e.s.d.'s in cell parameters are only used when they are defined by crystal symmetry. An approximate (isotropic) treatment of cell e.s.d.'s is used for estimating e.s.d.'s involving l.s. planes.
Refinement. Refinement of *F*^2^ against ALL reflections. The weighted *R*-factor *wR* and goodness of fit *S* are based on *F*^2^, conventional *R*-factors *R* are based on *F*, with *F* set to zero for negative *F*^2^. The threshold expression of *F*^2^ > σ(*F*^2^) is used only for calculating *R*-factors(gt) *etc*. and is not relevant to the choice of reflections for refinement. *R*-factors based on *F*^2^ are statistically about twice as large as those based on *F*, and *R*- factors based on ALL data will be even larger.

### Fractional atomic coordinates and isotropic or equivalent isotropic displacement parameters (Å^2^)

**Table:**

	*x*	*y*	*z*	*U*_iso_*/*U*_eq_	
O1	0.6259 (4)	0.9009 (4)	−0.2515 (2)	0.085 (2)	
O2	0.5311 (3)	0.7583 (2)	0.08959 (15)	0.0374 (6)	
O3	0.7763 (3)	0.7304 (2)	0.10000 (19)	0.0431 (6)	
O4	0.6719 (5)	0.3876 (3)	0.0578 (3)	0.0835 (13)	
O5	0.5534 (3)	0.5637 (2)	0.30153 (17)	0.0495 (7)	
N1	0.8069 (4)	0.5351 (3)	−0.0253 (2)	0.0600 (11)	
H1A	0.8224	0.4786	−0.0701	0.072*	
H1B	0.8429	0.6155	−0.0279	0.072*	
N2	0.4593 (4)	0.7713 (3)	0.2737 (2)	0.0461 (8)	
H2A	0.4389	0.7854	0.3317	0.055*	
H2B	0.4395	0.8322	0.2324	0.055*	
C1	0.5709 (6)	0.7727 (7)	−0.2635 (3)	0.093 (12)	
H1C	0.5441	0.7602	−0.3280	0.140*	
H1D	0.4871	0.7613	−0.2245	0.140*	
H1E	0.6429	0.7069	−0.2466	0.140*	
C2	0.6605 (5)	0.9407 (5)	−0.1622 (3)	0.0582 (12)	
C3	0.7152 (6)	1.0709 (5)	−0.1530 (3)	0.0679 (15)	
H3A	0.7253	1.1252	−0.2059	0.081*	
C4	0.7541 (8)	1.1201 (4)	−0.0684 (4)	0.0788 (18)	
H4A	0.7932	1.2068	−0.0638	0.095*	
C5	0.7366 (6)	1.0431 (4)	0.0109 (3)	0.0581 (12)	
H5A	0.7614	1.0782	0.0692	0.070*	
C6	0.6820 (4)	0.9131 (3)	0.0036 (2)	0.0380 (8)	
C7	0.6436 (4)	0.8611 (4)	−0.0833 (2)	0.0444 (9)	
H7A	0.6069	0.7735	−0.0883	0.053*	
C8	0.6654 (4)	0.8314 (3)	0.0910 (2)	0.0355 (8)	
H8A	0.6676	0.8918	0.1456	0.043*	
C9	0.5521 (4)	0.6389 (3)	0.1436 (2)	0.0334 (8)	
H9A	0.4904	0.5665	0.1186	0.040*	
C10	0.7109 (4)	0.6039 (3)	0.1246 (3)	0.0385 (8)	
H10A	0.7553	0.5695	0.1823	0.046*	
C11	0.7276 (5)	0.4985 (4)	0.0474 (3)	0.0469 (10)	
C12	0.5210 (4)	0.6565 (3)	0.2480 (3)	0.0349 (8)	

### Atomic displacement parameters (Å^2^)

**Table:**

	*U*^11^	*U*^22^	*U*^33^	*U*^12^	*U*^13^	*U*^23^
O1	0.076 (2)	0.142 (3)	0.0376 (13)	−0.018 (2)	−0.0052 (15)	0.0087 (18)
O2	0.0403 (13)	0.0370 (12)	0.0349 (12)	−0.0019 (11)	−0.0030 (12)	0.0073 (11)
O3	0.0365 (13)	0.0359 (12)	0.0569 (16)	0.0001 (11)	−0.0015 (12)	0.0047 (12)
O4	0.128 (3)	0.0379 (16)	0.084 (2)	−0.0130 (19)	0.065 (2)	−0.0087 (16)
O5	0.0698 (18)	0.0423 (13)	0.0363 (13)	0.0070 (14)	0.0056 (14)	0.0088 (11)
N1	0.084 (3)	0.0436 (18)	0.053 (2)	−0.0035 (19)	0.038 (2)	−0.0072 (16)
N2	0.0574 (19)	0.0420 (16)	0.0390 (17)	0.0054 (17)	0.0148 (15)	0.0005 (13)
C1	0.065 (3)	0.165 (7)	0.049 (2)	−0.063 (4)	0.031 (2)	−0.035 (4)
C2	0.047 (2)	0.093 (3)	0.0348 (19)	0.010 (3)	0.0024 (18)	0.009 (2)
C3	0.091 (4)	0.053 (3)	0.060 (3)	0.021 (3)	0.029 (3)	0.023 (2)
C4	0.136 (5)	0.034 (2)	0.066 (3)	0.001 (3)	0.046 (3)	0.008 (2)
C5	0.088 (3)	0.036 (2)	0.050 (2)	−0.003 (2)	0.017 (2)	−0.0037 (18)
C6	0.044 (2)	0.0334 (17)	0.0366 (18)	0.0001 (15)	0.0056 (16)	0.0035 (15)
C7	0.043 (2)	0.052 (2)	0.0388 (19)	−0.0026 (18)	0.0046 (18)	0.0007 (18)
C8	0.0388 (18)	0.0318 (16)	0.0360 (17)	0.0027 (14)	−0.0012 (17)	0.0020 (15)
C9	0.0400 (19)	0.0285 (16)	0.0318 (17)	−0.0028 (15)	−0.0019 (16)	0.0024 (13)
C10	0.044 (2)	0.0333 (17)	0.0381 (18)	0.0026 (16)	0.0075 (16)	0.0042 (15)
C11	0.059 (3)	0.0342 (18)	0.048 (2)	0.0034 (18)	0.023 (2)	0.0064 (16)
C12	0.0405 (19)	0.0309 (16)	0.0332 (15)	−0.0022 (15)	0.0019 (16)	−0.0011 (15)

### Geometric parameters (Å, °)

**Table:**

O1—C1	1.372 (7)	C2—C7	1.380 (5)
O1—C2	1.371 (5)	C2—C3	1.385 (7)
O2—C8	1.434 (4)	C3—C4	1.350 (8)
O2—C9	1.419 (4)	C3—H3A	0.9300
O3—C8	1.434 (4)	C4—C5	1.371 (6)
O3—C10	1.429 (4)	C4—H4A	0.9300
O4—C11	1.217 (5)	C5—C6	1.380 (5)
O5—C12	1.228 (4)	C5—H5A	0.9300
N1—C11	1.320 (5)	C6—C7	1.387 (5)
N1—H1A	0.8600	C6—C8	1.492 (5)
N1—H1B	0.8600	C7—H7A	0.9300
N2—C12	1.318 (4)	C8—H8A	0.9800
N2—H2A	0.8600	C9—C12	1.527 (5)
N2—H2B	0.8600	C9—C10	1.531 (5)
C1—H1C	0.9600	C9—H9A	0.9800
C1—H1D	0.9600	C10—C11	1.521 (5)
C1—H1E	0.9600	C10—H10A	0.9800
			
C2—O1—C1	117.8 (4)	C7—C6—C8	121.4 (3)
C9—O2—C8	106.9 (2)	C2—C7—C6	119.3 (4)
C10—O3—C8	109.0 (3)	C2—C7—H7A	120.3
C11—N1—H1A	120.0	C6—C7—H7A	120.3
C11—N1—H1B	120.0	O2—C8—O3	105.6 (2)
H1A—N1—H1B	120.0	O2—C8—C6	110.4 (3)
C12—N2—H2A	120.0	O3—C8—C6	112.1 (3)
C12—N2—H2B	120.0	O2—C8—H8A	109.6
H2A—N2—H2B	120.0	O3—C8—H8A	109.6
O1—C1—H1C	109.5	C6—C8—H8A	109.6
O1—C1—H1D	109.5	O2—C9—C12	114.2 (3)
H1C—C1—H1D	109.5	O2—C9—C10	102.8 (3)
O1—C1—H1E	109.5	C12—C9—C10	112.2 (3)
H1C—C1—H1E	109.5	O2—C9—H9A	109.1
H1D—C1—H1E	109.5	C12—C9—H9A	109.1
O1—C2—C7	124.7 (5)	C10—C9—H9A	109.1
O1—C2—C3	115.9 (4)	O3—C10—C11	112.0 (3)
C7—C2—C3	119.4 (4)	O3—C10—C9	104.6 (3)
C4—C3—C2	120.9 (4)	C11—C10—C9	112.3 (3)
C4—C3—H3A	119.5	O3—C10—H10A	109.3
C2—C3—H3A	119.5	C11—C10—H10A	109.3
C3—C4—C5	120.5 (4)	C9—C10—H10A	109.3
C3—C4—H4A	119.7	O4—C11—N1	125.1 (4)
C5—C4—H4A	119.7	O4—C11—C10	118.8 (3)
C4—C5—C6	119.6 (4)	N1—C11—C10	116.0 (3)
C4—C5—H5A	120.2	O5—C12—N2	124.9 (3)
C6—C5—H5A	120.2	O5—C12—C9	118.4 (3)
C5—C6—C7	120.2 (3)	N2—C12—C9	116.7 (3)
C5—C6—C8	118.4 (3)		
			
C1—O1—C2—C7	−0.1 (7)	C5—C6—C8—O3	−104.0 (4)
C1—O1—C2—C3	−179.8 (5)	C7—C6—C8—O3	76.1 (4)
O1—C2—C3—C4	−179.3 (5)	C8—O2—C9—C12	88.2 (3)
C7—C2—C3—C4	1.0 (8)	C8—O2—C9—C10	−33.7 (3)
C2—C3—C4—C5	−1.7 (9)	C8—O3—C10—C11	114.2 (3)
C3—C4—C5—C6	1.6 (8)	C8—O3—C10—C9	−7.6 (4)
C4—C5—C6—C7	−0.7 (7)	O2—C9—C10—O3	25.2 (3)
C4—C5—C6—C8	179.5 (5)	C12—C9—C10—O3	−98.0 (3)
O1—C2—C7—C6	−179.7 (4)	O2—C9—C10—C11	−96.5 (3)
C3—C2—C7—C6	−0.1 (6)	C12—C9—C10—C11	140.3 (3)
C5—C6—C7—C2	−0.1 (6)	O3—C10—C11—O4	−177.2 (4)
C8—C6—C7—C2	179.8 (4)	C9—C10—C11—O4	−59.9 (5)
C9—O2—C8—O3	29.8 (3)	O3—C10—C11—N1	4.8 (5)
C9—O2—C8—C6	151.1 (3)	C9—C10—C11—N1	122.1 (4)
C10—O3—C8—O2	−12.7 (3)	O2—C9—C12—O5	−171.1 (3)
C10—O3—C8—C6	−132.9 (3)	C10—C9—C12—O5	−54.6 (4)
C5—C6—C8—O2	138.5 (4)	O2—C9—C12—N2	9.5 (5)
C7—C6—C8—O2	−41.3 (4)	C10—C9—C12—N2	126.1 (3)

### Hydrogen-bond geometry (Å, °)

**Table:**

*D*—H···*A*	*D*—H	H···*A*	*D*···*A*	*D*—H···*A*
N2—H2B···O5^i^	0.86	2.33	3.076 (4)	145
N2—H2A···O4^i^	0.86	2.13	2.926 (4)	153
N1—H1B···O2^ii^	0.86	2.31	3.045 (4)	143
N1—H1A···O5^iii^	0.86	2.20	2.952 (4)	146
C9—H9A···Cg1^iv^	0.98	2.82	3.640 (4)	141

Symmetry codes: (i) −*x*+1, *y*+1/2, −*z*+1/2; (ii) *x*+1/2, −*y*+3/2, −*z*; (iii) −*x*+3/2, −*y*+1, *z*−1/2; (iv) −*x*, *y*+1/2, −*z*+3/2.
